# Supplementary figures and images for: Lymphocyte activation after a high-intensity street dance class
Source: PLoS One. 2020 Sep 21;15(9):e0239516. doi: 10.1371/journal.pone.0239516 (PMC7505442; doi:10.1371/journal.pone.0239516)

**Supporting information**

**S1 Fig**

**A B**

**
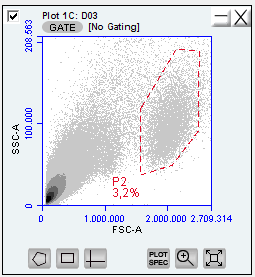

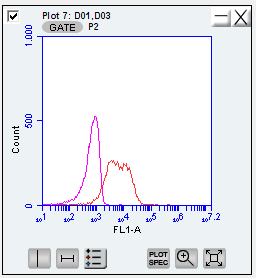
**

Supplement: S1 Fig — Lymphocytes of interest were gated according to forward and sideward scatter (FSC/SSC) (A). The histogram illustrates the negative control on the left (pink) and the sample with fluorescent on the right (red) (B). (DOCX) [file pone.0239516.s001.docx]
