# Supplementary material for: Lymphocyte activation after a high-intensity street dance class
Source: PLoS One. 2020 Sep 21;15(9):e0239516. doi: 10.1371/journal.pone.0239516 (PMC7505442; doi:10.1371/journal.pone.0239516)
Supplement: Attachment — Submitted filename: Response to Reviewers_PONE-D-20-17618.doc [file pone.0239516.s002.doc]

**COMMENTS TO REFEREES:**

We thank the reviewer for all points raised. We improved the manuscript by considering your comments. All modifications, made in the manuscript, are highlighted with tracked changes.

**Reviewer #1:**

MAJOR Comments.

- The authors are cautioned to not overstate their results. Most importantly, the conclusion that the bout of exercise led to transient immunosuppression should be struck from the manuscript (line 39-40, line 177-181, 196-197), as the authors have no supporting evidence that any of the dancers had suppressed immune systems.

Response:

We removed the following statements:

*- “These data may be useful to plan strategies to protect dance practitioners against microorganism infection and chronic inflammation”* (page 2, lines 40-41).

- *“Since changes in cell surface adhesion molecule expression on leukocytes are commonly associated with lymphocyte homing propensity [13] and L-selectin seems to be predominantly involved in tissue-specific lymphocyte homing [15], our data suggest that a single bout of street dance class may lead in disruption to homing mechanisms and elevation in adhesion molecules”* (page 9, lines 196-200).

- *“our data demonstrate that immediately after a street dance class, lymphocytes from dancers were less responsive to stimuli, thereby indicating transient immunosuppression.”* (page 11, lines 234-236).

- *“These findings may be useful to plan strategies to protect dance practitioners against microorganism infection and chronic inflammation.”* (page 11, lines 238-240).

- *“Thus, these events point to the decreased efficiency of lymphocytes against infection, when exposed to pathogens, after the street dance training (Fig 5)”* (page 10, lines 216-218).

- Figure 5 should also be removed, as there is no evidence provided of chronic inflammation, joint destruction, or arthritis.

Response: Figure 5 was removed.

- The results here do not show changes at a single cell level, and so again data must be interpreted with caution. The paragraph beginning on line 165 should be carefully rewritten.

Response: The authors rewrote the paragraph and briefly discussed the redistribution of CD62L- lymphocytes into the blood after exercise. We added on page 9-10, lines 200-208:

*“CD62L ensures that lymphocytes migrate from the blood into the lymph tissues [6] and research suggests that the reduction in CD62L+ lymphocytes after intense exercise may be due to the redistribution of CD62L- lymphocytes into the blood [15]. Moreover, Nielsen et al. noted a lower expression of CD62L after a marathon, and this reduction was followed by higher values of soluble CD62L, suggesting a shedding of adhesion molecules [16]. Since the different functions of these aspects during the regeneration phase are not fully understood, a clinical perspective of these alterations, such as the occurrence of infections after exercise, is only hypothetical and further research is crucially needed on this matter.”*

Methods.

-What formula was used to estimate maximum HR?

Response: For calculation of HR maximum, we used the formula: HR maximum = 220 – age. We added this information on page 4, lines 87-88.

- It would be useful to show actual HR at various intervals throughout the exercise session.

Response: The HRR was measured at 20, 40, and 60 minutes after the class. The authors added the reviewer suggestion in the discussion section, please see page 11, lines 231-233:

*“Also, the intensity of street dance training in future studies should investigate the HR reserve at different intervals throughout the exercise session.”*

- 85-89% of HRR does not seem particularly intense, particularly when considering the young adults exercised ~16 hours a week.

Response: The training intensity was classified as intense according to the guidelines of the American College of Sports Medicine (Tenth edition) (information added on page 4, lines 91-93).

-Why were only men included?

Response: the hormonal variations of the menstrual cycle modify the thermoregulation response to exercise in females, such that the core temperature during rest and exercise may differ according to the phases of the menstrual cycle (Sports Med. 2011;41(10):861-82. doi: [10.2165/11593180-000000000-00000](https://pubmed.ncbi.nlm.nih.gov/21923203/)), a fact that could influence the data in this study by increasing the variation in results and the risk of bias, thus possibly requiring a larger sample size. Additionally, we noted a greater number of male dancers during the recruitment of the participants.

-What was the delay between cessation of exercise and the post-blood draw?

Response: The blood collection delay between participants was approximately 3-4 min. Information was added in methods (Page 5, line 104).

- What anti-coagulant was used? What were the inter- and intra- assay variations for CK and LDH determination?

Response: Blood samples were drawn into BD Vacutainer® tubes containing heparin (information added on page 5, line 103). The linear correlation coefficient for CK and LDH was 0.999 and 0.9992, respectively (information was added in methods - page 6, lines 124-125).

- Line 111-118 What reaction is catalyzed that can be measured with fluorescence?

Response: hydroethidine (a decreased derivative of ethidium bromide) is intracellularly oxidized by oxygen radicals, being converted into ethidium bromide that tightly binds to DNA and presents a strong red fluorescence. Information added on page 6, lines 131-134.

-Representative flow cytometry plot illustrating gating strategy is needed.

Response: The gating strategy was added in supporting information (please see page 8, lines: 177-178).

- Were all 30 samples assessed on a single day, or were they assessed on different days? If on different days, what strategies were used for calibration/standardization?

Response: On the day of the experiment, we calibrated the flow cytometer to confirm that the instrument was performing properly by tracking laser, detectors, and fluidic performance. This quality control was performed by fluorescent beads and according to the manufacturer's instructions (Becton Dickinson, CA, USA) (information added on page 6, line 134). All 30 samples were assessed on a single day and this information was added on page 5, lines102-103.

-Statistics: Paired (dependent) T-tests? Were assumptions of normality and variance met? Without this information the reader cannot understand if appropriate tests were applied.

Response: The authors previously performed the data normalization (Shapiro-Wilk test). Information added on page 7, lines 151-152.

Results:

-Where are the LDH results?

Response: LDH result was added as Figure 1E e described in the results section. Please see page 8, lines 158-159.

- Why is L-selectin not shown? How was L-selectin measured?

Response: This research is the sequence of a previous study and the plasma L-selectin value was presented in the manuscript (Title: Neutrophil Migration and Adhesion Molecule Expression after Acute High-Intensity Street Dance Exercise) published in 2018 in the journal Mediators of Inflammation. This information is stated on page 8, lines 173-174.

-How were lymphocytes counted? How were lymphocytes quantified?

Response: After isolation of blood lymphocytes (page 5, lines 101-116), samples were mixed with Türk’s solution (1:20) and the lymphocyte number was immediately measured using a Neubauer chamber under an optical microscope (Nikon, Melville, NY). The information was better described (please see page 5, lines 101-118).

MINOR comments:

Page 4

Line 76/77 - Both body weight and body mass listed, yet numbers are different.

Line 83 - Improper subject verb number agreement (“dancers dances”)

Line 88-91 - Description of exclusion criteria is long, yet vague. Please be more specific in regard to the rationale for the exclusion criteria.

Response: The authors corrected the manuscript and the change can be evidenced in line 78, line 86, and lines 95-99.

Page 5

Line 114- Just PMA, not PMA+Ionomycin?

Line 104 - How long were cells cultured?

Response: The authors used only PMA to determine ROS (incubated for 30 minutes). Information added on page 6, line 129. The cells were maintained in culture for 1 hour to enable the monocytes to adhere to the plates (information added – lines 114-115).

Abstract: define abbreviations at first use.

Line 153 - “Our data observed” – wrong verb

Line 153 - “single session [of] street dancing” (“of” not included)

Line 155 - “Besides” – awkward word choice. “In addition” might work better

Line 200 - The use of “dance practitioners” is awkward. Just use “dancers.”

Figure 1 - Are axes flipped on 1d? CK-MB scale does not match 1b.

- Figure 3: CD62[L] (“L” not included in axis) units should be the same on 3a and 3b, they are not currently.

- Figure 4: 16 data points on the graph but only 15 participants were in the study.

Figure 5: I suggest removal.

Line 281 - Remove “was analyzed” from first sentence of caption.

Response: The authors are grateful for the identified mistakes. All points were duly reviewed and corrected.

Figure 2

- Shouldn’t ROS be in fluorescence units (e.g. MFI)?

To assist visualization, the histogram of ten thousand events is shown in arbitrary units (logarithmic scale). The logarithmic scale information was added to the figure legend (lines 327-328).

**Reviewer #2:**

Introduction:

- Can the authors define their abbreviations within the manuscript (CK, LDH, etc.).

- Line 55-57: “sL-selectin…preventing the binding of leukocytes to those cells” Prevents the binding to which cells?

Response: Abbreviations have been defined and the phrase was revised (please, see page 3, line 58).

Methods:

- Line 75: “The dancer’s volunteers”: It is unclear what the authors are referring to here

- Blood counts provided in lines 79-82: it is not clear in the manuscript if these were resting values?

- For the correlations are the authors suggesting that each individual exercised at a different relative intensity? If so, this calculation needs to be better phrased.

Response: we made the adjustments as suggested. Blood count corresponds to the resting values and the correlations of the study were obtained from the HR reserve of each participant. The changes can be evidenced in lines 76 and 83-84 (page 4), and lines 94-95 (page 4-5).

- Measurement of reactive oxygen metabolites: Can the authors clarify what is being measured in the FL3 channel. Are the cells fluorescing and the signal is detecting in FL3?

Response: The hydroethidine (a decreased derivative of ethidium bromide) is intracellularly oxidized by oxygen radicals, being converted into ethidium bromide that tightly binds to DNA and presents a strong red fluorescence. This fluorescence is measured in the FL3 channel. Explanation of the method was described on page 6, lines 131-135).

Results/Discussion:

- Would the authors consider plotting all bar graphs in to show the raw data (individual data points) as well as the means/SEM?

Response: The authors plotted the data as requested by adding the graphs with raw data (individual data points) and they can be found as Supplementary Materials (Figure S1).

- Figure 5:

- Systemic is misspelled

- Immunosuppression infection risk is not clear. It reads as if chronic inflammation added with intense dancing would lead to joint destruction/arthritis. What the authors report in this study is an acute phase of inflammation immediately after exercise, not chronic inflammation found in these individuals.

Response: Thank you for the observation. Regarding Figure 5, the authors agree with the points raised by #reviewer1 and chose to remove the figure.

- Line 175: can the authors expand on the statement: the inflammatory pattern must be a self-controlled event?

Response: The statement was expanded and a classic example was added (please see page 10, lines 210-212).

- The authors state in their discussion that clinical consequences of their findings is not clear, yet they speculate without much evidence. Can the authors strengthen their argument for this.

Response: The authors agree with reviewers #1 and #2 regarding the precaution to not overstate the results. Thus, it was removed from the following statements:

*- “These data may be useful to plan strategies to protect dance practitioners against microorganism infection and chronic inflammation”* (page 2, lines 40-41).

- *“Since changes in cell surface adhesion molecule expression on leukocytes are commonly associated with lymphocyte homing propensity [13] and L-selectin seems to be predominantly involved in tissue-specific lymphocyte homing [15], our data suggest that a single bout of street dance class may lead in disruption to homing mechanisms and elevation in adhesion molecules”* (page 9, lines 196-200).

- *“our data demonstrate that immediately after a street dance class, lymphocytes from dancers were less responsive to stimuli, thereby indicating transient immunosuppression.”* (page 11, lines 234-236).

- *“These findings may be useful to plan strategies to protect dance practitioners against microorganism infection and chronic inflammation.”* (page 11, lines 238-240).

- *“Thus, these events point to the decreased efficiency of lymphocytes against infection, when exposed to pathogens, after the street dance training (Fig 5)”* (page 10, lines 216-218).
