# Supplementary material for: Lymphocyte activation after a high-intensity street dance class
Source: PLoS One. 2020 Sep 21;15(9):e0239516. doi: 10.1371/journal.pone.0239516 (PMC7505442; doi:10.1371/journal.pone.0239516)
Supplement: Attachment — Submitted filename: Response to Reviewers_PONE-D-20-17618R1.doc [file pone.0239516.s003.doc]

**COMMENTS TO REFEREES:**

The authors would like to thank the Academic editor for identifying this study as having

merit for publication in PLOS ONE. The authors also appreciate the critical comments provided by all the reviewers. We have worked to address all the comments and incorporate all the suggestions provided by all the reviewers. All modifications, made in the manuscript, are highlighted with tracked changes.

**Reviewer #1:**

* MEDIUM Comments*

- “It is unclear if CK, LDH, and L-selectin were measured from plasma or serum. The blood collection methods indicate blood collection into heparinized tubes (suggesting plasma analysis), yet enzymatic activity methods indicate using kits for serum activity determination. Furthermore, the text of the results indicates plasma analysis but the figure captions suggest serum. Which is it?”

- Lines 152/153: Serum/plasma issue (see medium comments).

Response: CK, LDH, and L-selectin were measured from plasma and this information was corrected (please see page 6, line 122; page 15, lines 325-326).

*MINOR Comments*

- Line 58: CD62L is mentioned without being introduced as an alternate name for L-selectin. It should be noted at this point or introduced as such prior.

- Line 66/67: It is not specified in what tissue the activities of CK and LDH were measured.

Response: The CD62L information was added on page 3, line 56. CK and LDH were measured from plasma (page 3, line 68).

- Line 64: “In the present [study], …”

- Line 84: (sentence fragment) “Monitored by Polar…” Sentence needs a subject. For example, “Heart rate (HR) was monitored continuously by Polar XXX heart rate monitors during exercise.”

- Line 90: “…any exercise for 72 hours before a dance class…” "a" should be replaced by "the"

- Line 96: Suggest that the statement regarding batch sample analysis be moved to line 98 following “...3-4 minutes.” as its own sentence for clarity.

- Line 98: “…and the blood collection delay [following exercise] between participants was approximately 3-4 minutes.”

- Line 122: Rephrase “The release of ROS was incubated for 30 minutes.” Perhaps: “The cells were incubated for 30 minutes prior to assessment of ROS production.”

- Line 125: "Reduced" rather than "decreased" is the appropriate chemical term in this instance.

- Line 164/169: Suggested that final sentence regarding gating strategy (line 169) be moved to line 164 to consolidate flow cytometry results.

- Line 174: “lymphocytes [in circulation].”

- Line 192: Suggest replacing “the regeneration phase with "recovery from exercise”

- Line 208: “molecules are..."Replace "induced" with "altered”

- Line 219: "In conclusion..." replace "pieces of evidence of" with ”elevated markers of"

- Line 220: “…suggesting [potential for] higher…”

- Line 221: “…process of leukocytes [following intense dance]."

Response: All grammatical and relocation suggestions were considered and can be observed at: page 3 (line 66), page 4 (lines 87-88, line 94), page 5 (lines 103-104, line 104), page 6 (lines 130-131, line 133), page 8 (lines 173-174), page 9 (line 182), page 10 (line 206, line 223), page 11 (line 236), page 11 (lines 238-239).

Figures:

- Generally, line graphs (Fig 1, 3, 4) should include R2 values associated with the line

- Figure 1: L-selectin is on different axes between 1c and 1d. This is a little confusing.

- Figure 4: X-axis STILL labelled CD62 not CD62L.

Response: The authors included R^2^ (Fig 1, 3, 4), L-selectin from Fig1c and Fig1d was positioned on the same axis, and Fig4 was labelled as CD62L.

**Reviewer #2:**

Only 2 MINOR comments:

- Bar Graphs: For transparency, I feel that all figures should be presented as scatter plot with bars and should be included in the main manuscript- however I will let the editor decide what is best.

Response: As suggested, all figures included in the main manuscript are presented as scatter plot (instead of bar graphs). Please see figures 1, 2, and 3.

- Lines 210-212 are unclear and very confusing. Please consider rephrasing or dropping the added sentences.

RESPONSE: The sentence was dropped (page 10, lines 224-226).
